# PATHLOGIC-S: A Scalable Boolean Framework for Modelling Cellular Signalling

**DOI:** 10.1371/journal.pone.0041977

**Published:** 2012-08-07

**Authors:** Liam G. Fearnley, Lars K. Nielsen

**Affiliations:** Australian Institute of Bioengineering and Nanotechnology, University of Queensland, Brisbane, Queensland, Australia; Semmelweis University, Hungary

## Abstract

Curated databases of signal transduction have grown to describe several thousand reactions, and efficient use of these data requires the development of modelling tools to elucidate and explore system properties. We present PATHLOGIC-S, a Boolean specification for a signalling model, with its associated GPL-licensed implementation using integer programming techniques. The PATHLOGIC-S specification has been designed to function on current desktop workstations, and is capable of providing analyses on some of the largest currently available datasets through use of Boolean modelling techniques to generate predictions of stable and semi-stable network states from data in community file formats. PATHLOGIC-S also addresses major problems associated with the presence and modelling of inhibition in Boolean systems, and reduces logical incoherence due to common inhibitory mechanisms in signalling systems. We apply this approach to signal transduction networks including Reactome and two pathways from the Panther Pathways database, and present the results of computations on each along with a discussion of execution time. A software implementation of the framework and model is freely available under a GPL license.

## Introduction

Rapid growth in the size of curated cellular signalling databases such as Reactome [1]–[4], Panther Pathways [5] and the NCI-Nature Pathway Interaction Database [6] have seen them approach or exceed the size of many common metabolic models. For example, there are 3,909 entities in the *Homo sapiens* metabolic model [7] compared to 6,504 entities in the *Homo sapiens* Reactome signalling model [1]. No current models of signalling demonstrate the ability to handle the kinds of complex, large scale systems that can now be generated from these and other sources. Instead, extant models focus on single signalling pathways such as the ATR pathway [8] or on small uncurated subsets of signalling databases [9]. Consisting of 322 signalling events acting on 526 chemical entities, the latter is less than 10% of the number of entities present in the Reactome database. Even randomly generated networks used to explore modelling approaches fall well short of genome scale with typical sizes being on the order of 150 signals with 50 signalling events [10].

The available databases can be used to define a model topology, i.e., a component list and their possible interactions. Dynamic models described with systems of ordinary differential equations [11] also require kinetic parameters to describe the rates of the identified interactions. While full parameterisation is generally feasible for smaller signalling models (at the individual pathway level) [12], parameterisation of signalling systems as they approach cellular scale is experimentally intractable. Parameterisation is avoided in constraint-based modelling. Genome-scale modelling of cellular metabolism is an accepted method of *in silico* hypothesis testing and experimental design, and is backed by a rich set of tools notably exemplified by the COBRA toolbox [13]–[15]. Such genome scale models are now routinely used to guide complex metabolic engineering designs [16], demonstrating that models relying solely on the topology of systems can produce valid and valuable information about system behaviour. Constraint-based modelling, however, is not immediately useful for signalling networks. The key set of constraints, namely material flux balances, has no equivalent for information flux through catalytic signalling cascades; stimulation of a single receptor may generate any number of activated downstream signals and amplification is in fact a key feature of signalling networks. Though a work-around has been developed [9], it produces substantial model overhead. Moreover, the magnitude of information fluxes in the framework has no physical meaning and hence the model does not present an advantage over simpler model formulations, such as Boolean logical statements.

In Boolean models, signals are activated when the necessary preconditions are met, and signal flow through the modeled system is traceable as a sequence of activated variables and statements. Boolean models can be implemented as logical hypergraphs [17], [18] and explored using graph theoretic techniques. Such approaches require exhaustive enumeration of possible input states in order to generate predictions for biological problems pertaining to redundancy. These techniques do not scale well, and are further hindered by the lack of an analogous concept to inhibition in graph theory, resulting in loss of tractability [17]. Some implementations of these graph-theoretic techniques are implicitly time parameterised [19], assuming that all signalling events take the same period of time and occur sequentially. These approaches (and related methods where the time parameterisation of reactions is explicitly set [8]) are viable in small systems. They have not been successfully applied to large-scale networks, in part due to the lack of availability of kinetic data in databases of signal transduction, and suffer from the same lack of tractability at scale as unparameterised graph theoretic techniques.

Integer programming implementations do not suffer these issues to the same extent, and certain formulations of these types of model are provably scalable and solvable in linear time [10], [20]. However, the implementation of inhibition needs to be approached with care in these systems. If approached from the perspective of Boolean logic, the presence of an inhibitor results in the inactivation of a signal. Using this approach can result in widespread model infeasibility, and the current methodology used to deal with this problem consists of selectively ignoring inhibition where it would cause logical incoherence [10], a process which is impractical at cellular scale.

We propose a non-parametric Boolean model framework using integer programming techniques and which models inhibition in a manner analogous to its physical mechanism. This framework uses inhibiting signals to prevent signalling events taking place rather than as deactivators of active signals, thus reducing model infeasibility. The intended purpose of the framework is to enable analysis of complex systems in terms of generation of network states given specified inputs and internal signal states. It does not model reaction kinetics and dynamics within the Boolean update rules, but enables users to model these through use of its curation subsystems to generate the appropriate Boolean network. Approaching the problem in this way allows us to rapidly compute and interrogate large models (such as Reactome or the NCI-Nature Pathway Interaction Database).

We present here both the specification and an implementation of this framework, along with analysis of data obtained from major large public databases using this technique. The implementation provided is capable of generating hypotheses about and prediction of biological function using data specified in the BioPAX format. Such hypotheses include (but are not limited to) those concerning network states when signals are set to specific states, and quantification of the number of alternative methods of generating a given state. Additionally, the software supports visual editing and curation of network topologies through the use of Cytoscape and other graph-visualisation tools, and features an automated strongly connected component detection and visualisation system. The software has been designed to efficiently run on modern desktop computers, and requires only the Java virtual machine and its included libraries (provided under various open-source licenses). It is freely available under a GPL open-source license.

## Results and Discussion

PATHLOGIC-S comprises two distinct parts - a problem specification that describes the formulation of a system of Boolean statements logically equivalent to a given signalling system, and the implementation of such in a desktop application. The PATHLOGIC-S application in its current form is primarily intended to quickly generate viable, testable hypotheses concerning the behavior of complex signalling systems using a standard desktop workstation. The implementation provided has been designed to deal with a number of questions, including determining the minimum input signals that must be active in order to produce a given network state (the Minimum Input problem), and counting how many non-trivial different sets of inputs give rise to a given network state (the Minimal Input Sets problem). It has been tested and developed on an Intel Core i7-820 CPU (four cores at 2.93 Ghz) with 8 Gb of RAM with the 64-bit version of the Windows 7 operating system, which was also used to generate result data for this work.

### The PATHLOGIC-S Specification

A given system of signals consists of a set of *n* physical entities, 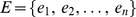 comprised of proteins, complexes, small organic molecules, and RNAs involved in signal transduction. These entities are involved in reactions referred to as signalling events that result in the transduction of information from one set of ‘active’ *signalling molecules* to another. The sets of physical entities and the signalling events that they take part in are sourced from databases of signal transduction.

We assign a set of *n* logical variables 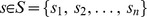 representing signal states in direct bijection with their physical entity equivalents (ie, the *i*th physical entity, 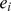, is represented by 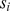 in the logical formulation).

We then represent each signalling event as a pair of Boolean expressions written as a sequence of AND (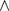) operators with a single IMPLIES (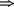) operator on a set of *m* variables 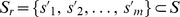 containing both input and output signals, and a reaction variable *r*. The resultant Boolean expressions, *B*, are of the form 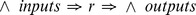.

We then rewrite *B* to produce a new set of statements *B’*, in which each statement is either of the form 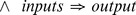 or 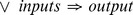. If the statement is one showing the activation of a reaction variable (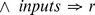), then no change is necessary and the statement is added to *B’* without modification. We then iterate through the remainder of the statements, creating a set of statements about which signalling events give rise to each individual output signal. These statements are of the form 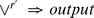 (where *r’* are the reactions giving rise to a specific *output*), and are added to *B’*.

Unique to our formulation is the method of adding information about inhibition (or repression) of signal to *B’*. Inhibition is produced in a Boolean system using the NOT operator (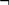) and is total in nature – there is no partial inhibition possible in a standard Boolean representation. In the PATHLOGIC formulation, inhibition is only modeled as being applied to signalling events rather than to the signals themselves. Thus, if a signalling event (with corresponding activating Boolean expression 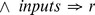) is inhibited by some set of inhibitors, we add these inhibitors to its Boolean representation so that it becomes 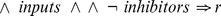. If an inhibitor is general rather than reaction specific (ie, it prevents signalling by that entity), we add the inhibitor to all signalling events resulting in production of that signal.

**Figure 1:**
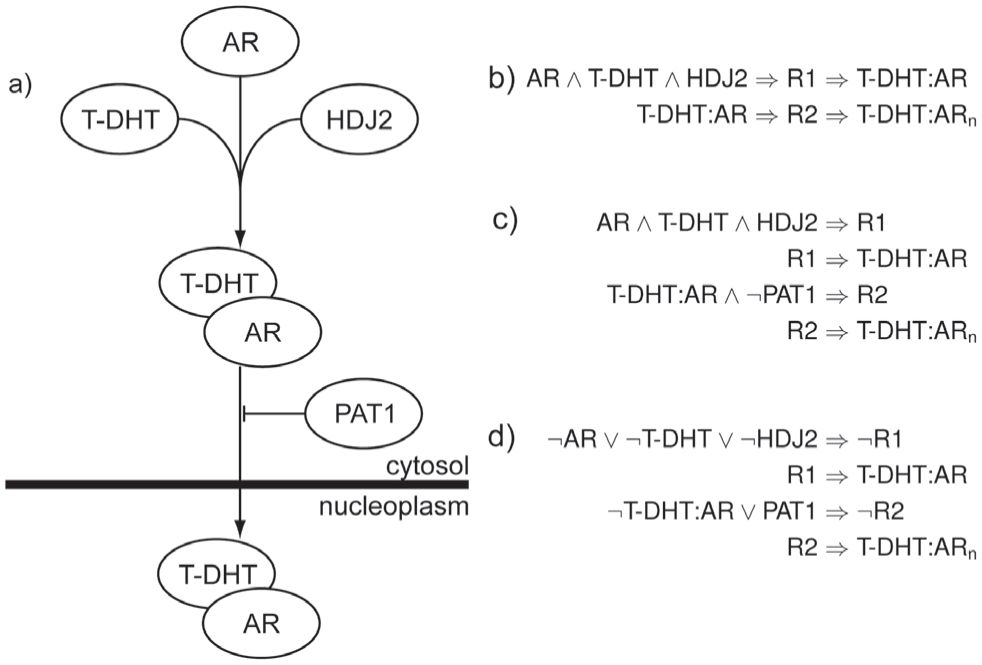
An example of a PATHLOGIC-S formulation. **a)** A pair of reactions from the androgen receptor pathway of the NCI-Nature Pathway Interaction Database [6], [35]–[38]. Inhibiting interactions are presented with a flat-ended arrow. **b)** Initial logical formulation. Conversion of upstream signals, catalysts, and activating signals takes place at this point. **c)** Logical statements with inhibition information added, prior to conversion to disjunctive form. **d)** Logical statements in disjunctive form prior to conversion to a system of linear constraints.

Once this has been completed, the system is then rewritten in a conjunctive normal form and translated into a set of integer constraints as described in [10] and illustrated in the supplementary material (Methods S1). An example of this process on example data is contained in Figure 1.

This representation of inhibition prevents major problems with the state of signals being undefined and indeterminate in the logical statements. Such issues have been seen in other logical models using integer programming [10]. For instance, consider two signals, *S1* and *S2*. *S1* activity results in *S2* activity, but this is inhibited by the inhibitor *I*. Extant approaches allow this situation to be formulated as:


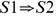
(1)


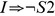
(2)

These statements are evaluated simultaneously by the integer programming method. Functionally, this will present the expected result when *S1* is active and *I* is inactive, in that *S2* will be produced. However, the inactivity of *I* will result in the activity of *S2* even in the absence of *S1* (as by logical transposition, 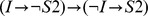), and when *S1* and *I* are active, the state of *S2* is indeterminate (it is true by the statement 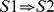, and false by 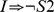). The PATHLOGIC-S formulation produces the correct behaviour, where the absence of the inhibitor is not sufficient to activate the inhibited signal, and the simultaneous presence of inhibitor and activator does not generate an infeasible logical state. This reduces overall logical infeasibility resulting from inhibition, avoiding many of the issues outlined in the introductory material.

### Validation and Hypothesis Generation

In order to show that the PATHLOGIC-S framework is capable of generating valid and testable hypotheses from data, we reconstructed a previously analysed, experimentally validated model of T-cell activation [8]. The model was instantiated according to the PATHLOGIC-S specification (supplied in Methods S1). The previous work had predicted the possibility of activation of Jnk (UniProt:P45983) without activation of Erk (UniProt:P27361), a hypothesis that contradicted prior experimental results [21]–[23]. This prediction was validated experimentally, and attributed to signal transduction resulting from availability of active CD28 (UniProt:P10747) as a signal input through Vav1 (UniProt:P15498) and Rac1 (UniProt:P63001) [8].

We repeated this experiment *in silico* by instantiating a minimal input set problem where the signal states of Jnk and Erk were specified as active and inactive respectively. Each member of the resulting minimal input set represents a combination of signal inputs that will give rise to this network state (Jnk active, Erk inactive). The results of the computation generated a two member set. The first solution describes the CD28 and Rac1(UniProt:P63000) induced activity experimentally demonstrated in the original experiment. The second solution presents a novel input combination of a ligand of the T-cell receptor (InterPro:IPR021663) (either as a peptide-MHC complex or antibody) and Lck (UniProt:P06239). Our model predicts signal proceeding through activation of Fyn (UniProt:P06241) to generate phosphorylated T-cell receptor and Abl (UniProt:P00519), which result in Zap70 (UniProt:P43404) mediated activation of the Lat (UniProt:O43561) signalosome. This solution describes Jnk activity in the event of T-cell activation (through the T-cell receptor). It has been experimentally demonstrated that CD28 is not required for Jnk activation in activated T-cells [24], [25], and CD28 is in fact inactive in our solution. Further, it has been shown in B cells that CD45- (UniProt:P08575) cells cannot activate Erk (CD45 is not active in our solution), and that Erk and Jnk activation is decoupled [26]. We suspect that the T-cell signalling model described in [8] has a number of default signal input activities that cause the model to fail to encounter certain network states. The use of optimisation techniques in our model avoids problems of initial parameterisation of this type. This lends further weight to the viability of our second solution as a hypothesis, presenting an interesting possibility for experimental validation.

### Network Statistics

We performed analysis on two broad categories of data - single-pathway networks (the Apoptosis and T-cell receptor networks of the Panther Pathways dataset), and a large network (Reactome). Single-pathway networks differ fundamentally from larger networks in that the curators of these pathways have defined the network with a view to limiting the system described to that controlling a specific biological function. As such, the single pathway networks lack features such as crosstalk (where a signal enters one functional pathway and crosses to the output of another) which increase the connectivity and complexity of signalling networks.

In contrast, networks generated from larger databases partially capture these phenomena but are limited by the fact that they are generally amalgams of functionally defined pathways. The fact that these networks are both larger and more complex is evident in the higher maximum indegree and outdegree (the number of reactions in which a species is an input (output, respectively)) for the Reactome compared to the Panther Pathways data. A sizable number of signals in Reactome take part in multiple pathways (such as EGFR, which participates in 5 pathways, or SOS1 which participates in 16), representing the points at which signal transduction crosses pathways and crosstalk occurs.

Interestingly, the majority of system outputs in each data set analyzed have a unique combination of system inputs (60–76.9% of system outputs with a minimal input set size of 1) leading to their activation (Table 1). While the proportion of individual outputs falling into this category in the Reactome data is similar to that seen in single pathway data, the Reactome minimal input sets are larger (30% of multiple element minimal input sets having more than 10 distinct inputs). This is likely due to the single pathway data capturing some (but not all) of the redundancy present for each system output, resulting in the presence of a minimal input set size for a given output which is greater than 1, but smaller than that seen in Reactome data.

**Table 1:** Comparison of Signalling Data.

	Average Min Input (Median)	Median Minimal Input Set	% Outputs With MIS >1
Reactome	3.35 (2)	1	23.3
Panther Apoptosis	9.2 (9.5)	1	40
Panther T-Cell Activation	7 (5)	1	28.57
Panther Apoptosis (Curated)	6 (4)	1	23.08
Panther T-Cell Activation (Curated)	10.25 (10)	1	25

Comparison of data sets used in study showing the the average minimum input for a single output, and average size of the minimal input set generated for each single output. A majority of outputs in each dataset assessed had a single activating input combination, with a sizable minority possessing multiple distinct activating inputs in all data sets analyzed.

**Figure 2:**
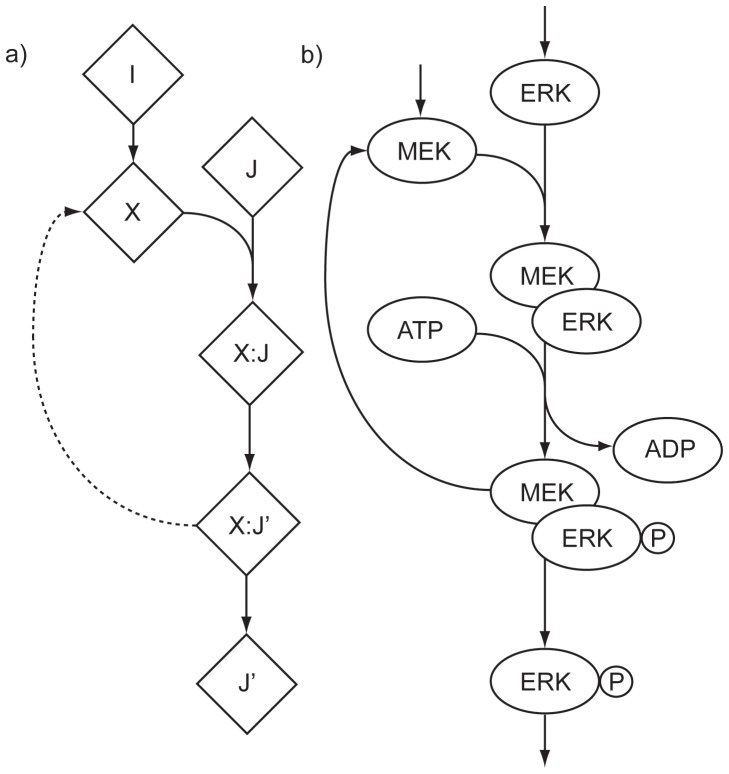
Logical loop structure, with an example from Reactome. **a)** An example of a logical loop structure. There exist two satisfying assignments to this set of statements when J’ is set to 1 - one with all variables except I set to 1, and one with all variables set to 1. To exclude the former solution from the solution space, the statement describing the relationship represented as a dashed line is removed from the logical formulation. **b)** In one mechanism of ERK phosphorylation, MEK and ERK are activated by various upstream processes (inputs). These active signals then form a complex, with MEK acting as a catalytic subunit of the complex resulting in ERK phosphorylation. The complex dissociates post-phosphorylation to yield phosphorylated ERK and MEK. Conversion to the logical form of this reaction yields a set of logic statements with associated SCC that has topology similar to **a)**.

The process of strongly connected component (SCC) removal (detailed in Methods) involves removal of redundant biological data associated with dissociation events and catalysis (Figure 2). This process can identify otherwise obscured system inputs and system outputs in the data (such as Bim (UniProt:O43521), which catalyses dissociation of a Bax-Bak complex (UniProt:Q07812, UniProt:Q16611) in the Panther Apoptosis data and becomes a system output post-curation), and also modifies the requirements for activation of certain signalling events.

As shown in Tables 2 and 3, SCC removal significantly modifies the topology of the network and as a result causes changes in both activation prerequisites and in the number of possible ways to activate given system outputs. This is attributable to the exclusion of satisfying assignments to the Boolean system that are not biologically valid (for example, the strongly connected components illustrated in the Methods) and the creation of new system inputs and outputs during the SCC removal process. This quantifiable change in the computed properties of the data clearly illustrates the importance of SCC removal in application of computational techniques to biological data.

**Table 2:** Data Properties and Statistics.

	Reactome	T-cell	Curated	Apoptosis	Curated
			T-cell		Apoptosis
Order	9838	150	147	192	188
Size	11139	161	151	234	224
Maximum Indegree	54	5	5	7	5
Maximum Outdegree	22	3	3	7	7
Number of Sinks	1851	13	14	10	13
Number of Sources	2909	45	47	68	68
Number of Strongly Connected Components	150	5	0	2	0
Average Number of Neighbours	2.24	2.12	2.05	2.44	2.38
Characteristic Path Length	24.07	9.69	8.63	6.43	4.81
Network Diameter	86	28	26	16	14
Network Radius	1	1	1	1	1

The nature of the topology is best described by taking measurements of its representative graph (formulated as discussed in the Curation section of the Methods). Curation and SCC removal (by its nature) reduces connectivity within the graph, leading to the observed differences between the curated/uncurated pairs. Reactome, by contrast, is a much larger dataset with much greater complexity, as shown in number of connected components and the characteristic path length.

**Table 3:** Analysis of Panther Pathways data.

	Minimum Input		Minimal Input Sets	
	Curated	Uncurated	Curated	Uncurated
**Apoptosis**				
Caspase 9	4	19	1	9
Bim	1	–	1	–
ATF	11	11	6	6
Bcl-2:Bik	2	2	1	1
NF 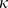 B	1	1	1	1
Bax:Bak	5	–	1	–
Fos:c-Jun	14	14	1	1
Bid	9	–	9	–
ELF2*α*	9	8	19	10
Degraded a127	13	13	1	1
endoG	3	18	1	9
I 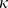 B:NF 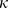 B	2	2	1	1
P53	4	4	1	1
**T-cell Activation**				
I 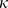 B	10	11	1	1
NF- 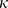 B	10	11	1	1
Pi	3	2	3	3
CRAC	10	–	1	–
jun:fos	23	12	6	6
cdc42:GTP:WASP	7	5	1	1
rac:GTP:Pak	7	5	1	1
NF-AT	12	3	1	1

Output signals from the apoptosis and T-cell activation pathways described in [5] are shown with the minimum number of input signals required for activation, and the number of (non-trivial) input combinations that give rise to the given output. Certain outputs are created in the curation process (eg, Bim) and thus lack results in the uncurated data.

### Performance

The software uses integer programming for network simulation under various conditions. Integer programming algorithms are highly efficient methods of addressing the problems presented here [27] and even for the largest data set (Reactome), a single instance of the problem can be solved in 60 ms on average. Writing output for visualization in Cytoscape [28] or Graphviz [29] requires additional time dependent on speed and type of disk drive – on a slow (5400 rpm) drive, this tends to be around 1300 ms, for a total per-problem execution time of approximately 1500 ms.

The time to perform analyses increases when characterizing networks using the Minimal Inputs and Minimal Input Sets problem definitions, due to the nature of the problems. Rather than solving for single instances of problems, these analyses require multiple instances to be rapidly addressed. In the case of minimal input sets, a naive, brute force approach to the problem consists of generating all possible input combinations then testing to determine whether or not the input gave rise to the network state of interest. Once the set of inputs giving rise to the desired activity had been elucidated, it could then be refined so that its members met the distinctness and minimality criteria described in the problem formulation. However, such an approach is intractable in networks larger than that of a single or double pathway, even when they are less than cellular scale in size. In the case of the Reactome data, which contains 2909 inputs, the naive approach requires 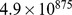 integer problems to be solved for each network state of interest.

Clearly, this naive approach is intractable, and an alternative enumeration and pruning strategy is required to reduce the number of instances of integer programming problems that must be solved. The PATHLOGIC-S implementation is as follows. We begin by solving the Minimum Input problem for the network state, in order to generate the minimum set of inputs that must be active for the state to arise. We then produce a set of descendent problems that exclude active inputs from their solutions by forcing previously active signals to be inactive, solve these, and generate additional descendents recursively retaining prior cuts until no valid solutions remain. The method in which this is performed is important. One approach would be to generate the set of all cuts possible from a solution (ie, the powerset of cuts, less the empty set). In the case where the solution had 10 active inputs, this would generate 1024 descendent problems. While this approach considerably improves over the naive method, a more aggressive cut is still possible.

Assume that we have some input *i* in an optimal solution of size *n* that is absolutely necessary for the activation of an output. There are then 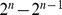 descendants that are guaranteed not to have solutions, as they contain a cut that results in the removal of *i* from the possible solutions. As such, instead of generating the powerset of cuts, we generate *n* single variable cuts from the solution that give rise to our descendent problems. The amount of computation that this saves is difficult to quantify precisely. In the case of Reactome, >75% of outputs have a minimal input set of size 1, and an average minimal input size of 3, which indicates that the use of individual cuts over powersets in generating descendents reduces the number of integer problems that must be formulated and solved by more than 11,000 instances.

Further, we perform a check to determine whether there are redundant outputs, where the set of incident edges on each output is identical. When such redundancy is encountered, we simply copy the output results for whichever output node is analyzed first, reducing the total number of computations with minimal initial precomputation.

In practical terms, we compute the minimal input sets for each individual output in Reactome. Using a brute force implementation as discussed earlier would require 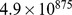 integer problems to be solved for each output to guarantee complete enumeration, disregarding the minimality and distinctness requirements. The implementation of the method described (single cut descendent generation with redundancy checks) on all 1851 outputs in the Reactome data requires the solution of only 662,099 integer problems, a feasible number for computation on standard desktop computers.

### Concluding Remarks

The PATHLOGIC-S specification handles inhibition of activity in a different way to other existing Boolean models of signalling [8], [10]. By representing inhibition as preventing processes that activate signals rather than as deactivating signals themselves, the model gains additional specificity and tractability. Practically, this lowers the amount of infeasibility due to model specification without the reduced solution space and resultant loss of information about biological function associated with other solutions to this problem.

PATHLOGIC-S does not time-parameterise individual signalling events. In trying to find logically satisfying assignments of Boolean variables, all events are effectively represented as taking place instantaneously. This results in PATHLOGIC-S generating a flat representation of oscillation that shows all possible signal activities across the states that comprise the cycle – an oscillation between two network states will be represented with all signals active in A and B being active in the PATHLOGIC-S solution. Additionally, there are some systems (such as that of Wnt/Erk [19]) that contain kinetic competition between signalling events. PATHLOGIC-S is not able to infer such behaviours automatically, but is capable of modelling them where explicitly stated either in the database as inhibition activities, or by a user in the curation process.

PATHLOGIC-S provides a rapid hypothesis generation function for model biological data in a common community exchange format (BioPAX), which runs on most modern desktops across a variety of operating systems. It has been deliberately designed to run on most modern computers using reasonable amounts of resources and time even on large datasets, thus avoiding the need for high performance computing to interrogate multiple pathways even when large amounts of crosstalk occur. Combined with the GUI and with the use of visualization tools for its output, we expect that PATHLOGIC-S will prove useful as a tool for studying the process of signal transduction even in large, complex signalling systems. PATHLOGIC-S is released as open-source under the GNU GPL license, and with the exception of JavaMail (released under the Sun JavaMail License as free software), all dependent libraries are licensed under the GNU GPL or Lesser GPL or Apache Software License, allowing for free use of PATHLOGIC-S outside of commercial software packages.

## Methods

### Optimization Problems for the PATHLOGIC-S System

We use and implement three key standard problems on the system – those of input minimization (or maximization), minimal input sets, and output maximization. These allow characterization of redundancy and elucidation of possible behaviors of the signalling system. We define *system inputs* to be any signals in the signalling network which are involved as inputs into an event but are not themselves outputs of any signalling event (including its activated events). Similarly, we define *system outputs* as any signals involved as outputs of signalling events but not used as input signals to any event (including its activating event). *Internal nodes* are defined as signals in the signalling network involved as an input to at least one signalling event, and as an output of at least one event.

The input minimization problem may be defined then as – for any set of system outputs/internal nodes assigned particular states, what is the minimal set of system inputs that give rise to a satisfying network state? This problem is instantiated by setting the bounds of the system outputs and internal nodes appropriately. If a signal is to be active, its corresponding logical variable is set to 1, and if it is to be inactive, it is set to 0. The objective function is then set to 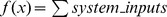, and the integer programming solver instructed to minimize (or maximize, if desired) 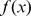.

A similar process is used in the output maximization problem. This is defined analogously to the input minimization, but in terms of assignments to system inputs, ie, ‘for any set of system inputs assigned particular states, what are all possible system outputs that can be generated?’. In the event that all inputs are specified, there is a single possible output set, which will be maximal. This problem is useful when partial knowledge of the input states is available, resulting in some inputs being left free for determination by the solver. Again, the problem is instantiated by setting bounds on variables (in this case system inputs), an objective function (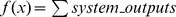) and maximizing 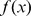.

Finally, we use the concept of a minimal inputs set to assess network redundancy. A minimal inputs set is defined as the set of sets of distinct minimal system inputs that give rise to the given output state. We define two minimal inputs (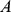 and 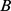) to be distinct if and only if there are some signals in *A* that are not in *B* and there are signals in *B* not present in *A* (ie, the relative complement of *A* to *B* is non-empty and vice versa). The minimal input set is enumerated using an iterative, sequential application of integer cuts.

Firstly, an integer programming problem is instantiated as per the input minimization problem, and solved to produce a minimal input set of size *j*. If this set is empty (ie, the output is constitutively expressed), the algorithm terminates. If 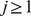 then *j* derivative problems are generated by copying the formulation of the prior integer programming instance but setting a system input that was active in the solution to be inactive. This process repeats until all minimal input sets are enumerated (ie, all derivative problems become infeasible and unsolvable), and the size of the minimal inputs set provides a quantitative metric for network redundancy.

### Model Curation and Strongly Connected Component Removal

A common feature of models of biological systems is the need for curation - the modification of the model in order to increase accuracy of predictions and allow internal consistency. These modifications are necessary due to the model formulations and are artefacts of the representation. For example, in flux-based models of cellular signalling, one aspect of curation covers the degradation of active signal once transduction takes place in order to maintain overall mass balance [9]. In logical models, the presence of strongly connected components (SCCs) or loops can cause erroneous predictions of input requirements. Consider the following toy example where *I* and *J* are inputs required for activation of *J’*, and the *:* character is used to show complexation of signal entities.


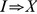
(3)


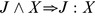
(4)


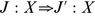
(5)


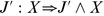
(6)

If the minimum input problem is instantiated for output *J’* in the prior set of logical statements, the result of 1 input required (namely, *J*) is returned by the algorithm. The reason for this is that when *J’* is set to active, there are two satisfying assignments to the statements. The first of these has all variables except *I* active, and the second (which is of interest) has all variables active. In order to fix this issue, we modify a single part of the network, as shown:


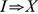
(7)


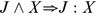
(8)


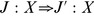
(9)


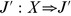
(10)

This removes the case where only *J* is required for activation of *J’*, and the desired behaviour is shown. To detect cases where this logical inconsistency might occur in the biological data, we take a set of statements derived from that data and reduce the problem to that of finding strongly connected components in a graph. We instantiate a graph, *G*, with vertices (*V*) and edges (*E*) such that each variable is assigned a unique node representation, and edges connect nodes 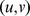 iff there is a logical statement where *u* is on the left side of an implies operator, and *v* on the right. We then execute Tarjan’s algorithm [30] on *G* to obtain the set of SCCs.

There are two common cases where these strongly connected components appear within the Reactome data, each associated with a biological process. The first arises when a SCC of order 2 is detected, and represents a case where catalysis is occurring. Such logical statements appear in the form 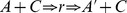. In order to obtain the desired behaviour in this case, the catalyst *C* is removed from the outputs, yielding the statement 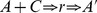. The second of these cases occurs when a set of proteins form a complex, one member of the complex is modified in some way, and the complex then dissociates. This requires a somewhat more complex fix, outlined in Figure 2.

Curation and SCC removal in PATHLOGIC-S is semi-automatic due to issues with fully automatic methods. For example, in Dasika et al [9], curation is automated by taking an input, performing loop detection using depth-first searching, disconnecting the edge that causes a loop to be formed and repeating until no further loops are encountered before moving to the next input. An example of how this strategy can fail is easy to see in the example presented in Figure 2. Application of this automated curation technique can result in the logical statement equivalent to one of two edges in the graph representation being deleted - 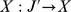, or 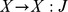 (if the search starts at node *I* or node *J*, respectively). If the 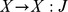 edge is deleted, the network allows formation of an *X:J* complex without the presence of *X*, which is undesirable. As such manual curation must be performed, and either be verified experimentally [10] or to be performed in such a way that it is consistent with the biological processes modelled (as discussed above). In PATHLOGIC, a graphical representation is generated for modification in Cytoscape [28], and then read back in after SCCs are removed, removing the logical statements from the system accordingly.

### Implementation Details

**Figure 3:**
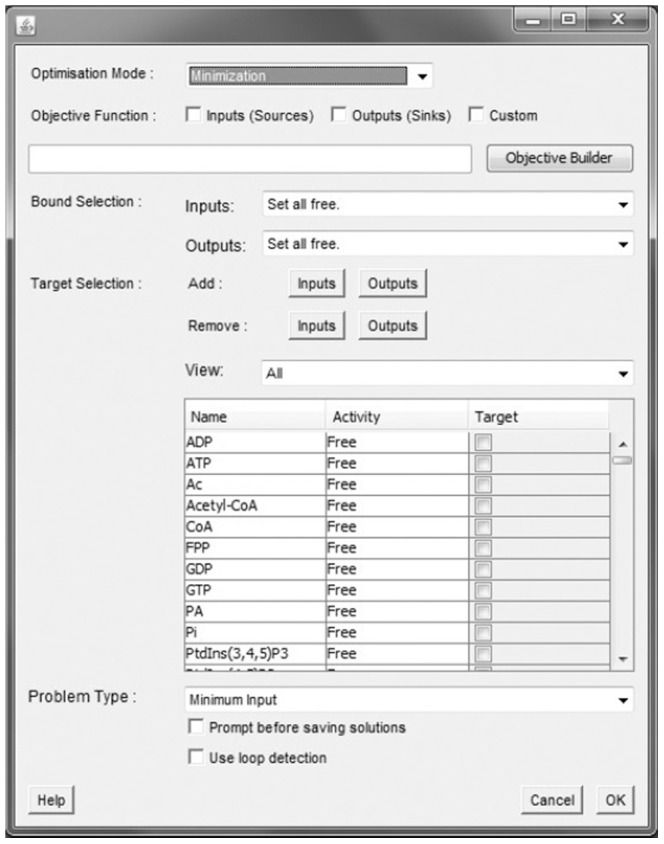
The main window of PATHLOGIC-S provides problem instantiation options. Custom objectives are supported through an objective builder, along with several presets. Target specification and activity settings are through the table presented, which can be restricted to show only system inputs and system outputs. At present, PATHLOGIC-S offers two methods of curation - one at the point of execution through dialog boxes, and one prior to execution through Cytoscape. Once the user clicks OK, the software executes the simulation, producing as output either a tab-delimited summary data file or a series of GML files specifying visualizations of the solutions found.

PATHLOGIC-S is implemented as a Sun Java [31] application written in the NetBeans IDE, and has been compiled and tested on Unix (AMD64 Debian Squeeze) and Windows 7 (64-bit and 32-bit). It uses the BioPAX Consortium’s paxtools library [32] for the input of BioPAX data and lpsolve [33] for integer programming capabilities. The software has a GUI implemented using Java Swing and AWT which allows users to set signal states (both input/output and internal nodes) and objective functions (Figure 3), and has the capability to notify users of job status via either email or through the Twitter microblogging service through use of the JavaMail and JTwitter libraries. By design, PATHLOGIC-S uses slightly less than 4 Gb of RAM (on large networks stuch as that generated from the Reactome data), allowing it to be run on most modern desktops once Java has been installed.

Implementation of the minimal input problem is relatively straightforward. A master problem consisting of the constraints formulated from the logical system and the objective function is generated. Individual problems are then instantiated using copies of this master problem, setting bounds for variables as required to perform computations. These individual problems are executed in parallel using the Java concurrency model and API for solving and output generation.

The implementation of the minimal input sets problem, however, is somewhat more complex due to the memory restrictions present in desktop computers. An initial optimization is necessary to generate the minimum possible set of inputs with some size 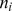. This minimal input set in turn allows the instantiation of 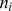 descendant problems as described above, and each of these descendant problems generate descendants in turn. At any given point, the PATHLOGIC-S application allows a user-defined number of threads to solve these problems. During the course of execution, the number of generated problems can (and likely will) exceed this limit. In the event that this occurs, the string representing the minimal input set that is generated as a result is stored for later expansion once execution of the currently live threads has completed.

Results output as plain text or GML files [34] for interpretation in Cytoscape or Graphviz. In these visualizations, the system of logical constraints is converted into a hypergraph representation (as it is depicted in biochemical diagrams), with information from the original database associated with each node. Nodes are then colored based on their logical variables −0, or false, represents the absence of the specified species, and a 1, or true, represents its presence.

The source code and pre-compiled binaries are available for download at the PATHLOGIC-S Sourceforge page and repository, at http://sourceforge.net/projects/pathlogic/.

### Biological Models

This study uses five key representations of biological models.

1. Reactome signalling data [1], defined as the complete Reactome dataset with the exclusion of certain pathways and entities listed in the supplementary material (Methods S2).
2. Apoptosis signalling data from Panther Pathways [5].
3. T-cell receptor signalling data from Panther Pathways [5].
4. Curated versions of the Apoptosis and T-cell signalling data from Panther Pathways [5].
5. A curated version of the T-cell receptor model [8].

Due to the changing nature of such data, archive files containing the specific files used in this study are enclosed as supplementary material (Methods S2).

## Supporting Information

Methods S1 (PDF)

Methods S2 (RAR)
