# Supplementary material for: PATHLOGIC-S: A Scalable Boolean Framework for Modelling Cellular Signalling
Source: PLoS One. 2012 Aug 7;7(8):e41977. doi: 10.1371/journal.pone.0041977 (PMC3413702; doi:10.1371/journal.pone.0041977)
Supplement: Methods S1 — (PDF) [file pone.0041977.s001.pdf]

Supplementary Material — Methods S1

Fearnley, Nielsen — PATHLOGIC-S: A Scalable Boolean Framework for Modelling Cellular Signalling

1 Formulation of Logic Model and Integer Constraints

The following example has been formulated to show catalysis, inhibition, multiple inputs/outputs, and alternative pathways activating the same signal. Each of these biological features of signalling networks appear frequently, but rarely in the close proximity illustrated in this artificial signalling network.

1.1 Chemical Reaction Formulation

We have a system composed of two signalling events, with signals S_A, S_B, a catalyst C, inhibitor I and outputs O_1, O_2:

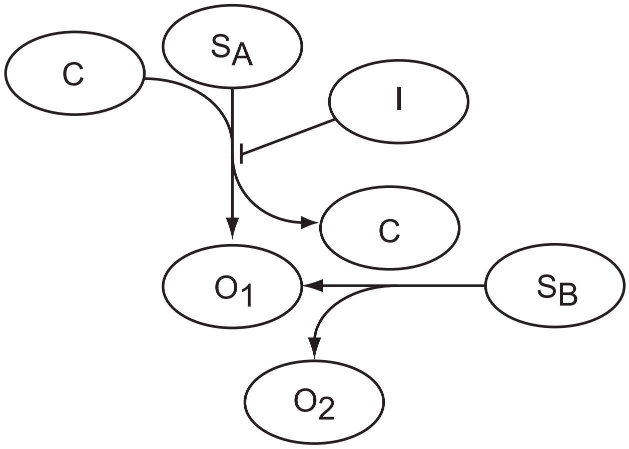

$$S_A + C \rightarrow O_1 + C \text{ inhibited by } I \tag{1}$$
$$S_B \rightarrow O_1 + O_2 \tag{2}$$

1.2 Step 1: Conversion to Logical Form

This is converted into a logical format (with variables labelled with the names of their equivalent signals for convenience) of :

$$S_A \wedge C \rightarrow R_1 \rightarrow O_1 + C \text{ inhibited by } I \tag{3}$$

$$S_B \rightarrow R_2 \rightarrow O_1 \wedge O_2 \tag{4}$$

The introduced intermediate variables (R_1 in this example) are important in the modelling of inhibition as shown in Step 3, and in the modelling of multiple different activating events.

1.3 Step 2: Conversion to Production Rules

The logical format must then be rewritten to produce a set of production rules for each species. Note that signals may appear on the right hand side of at most one logical statement in this form:

$$S_A \wedge C \rightarrow R_1 \text{ inhibited by } I \tag{5}$$

$$S_B \rightarrow R_2 \tag{6}$$

$$R_1 \rightarrow C \tag{7}$$

$$R_1 \vee R_2 \rightarrow O_1 \tag{8}$$

$$R_2 \rightarrow O_2 \tag{9}$$

1.4 Step 3: Integration of Inhibition Information

Information about inhibition must then be incorporated into the production rules using the NOT ($\neg$) operator. This changes equation 5 to:

$$S_A \wedge C \wedge \neg I \rightarrow R_1 \tag{10}$$

1.5 Step 4: Conversion to OR-form

The system of production rules is then converted into OR-form as per Haus (2009). The following system of statements results:

$$\neg S_A \vee \neg C \vee I \rightarrow \neg R_1 \tag{11}$$
$$S_B \rightarrow R_2 \tag{12}$$
$$R_1 \rightarrow C \tag{13}$$
$$R_1 \vee R_2 \rightarrow O_1 \tag{14}$$
$$R_2 \rightarrow O_2 \tag{15}$$

1.6 Step 5: Curation

Data presented to the system may be curated, a process that can be described as follows:

First, the system is converted to a graph formulation as discussed in the Materials and Methods. Each logical variable is represented as a node, and edges correspond to implies operators. For example, the logical statements described in Step 3 are presented as:

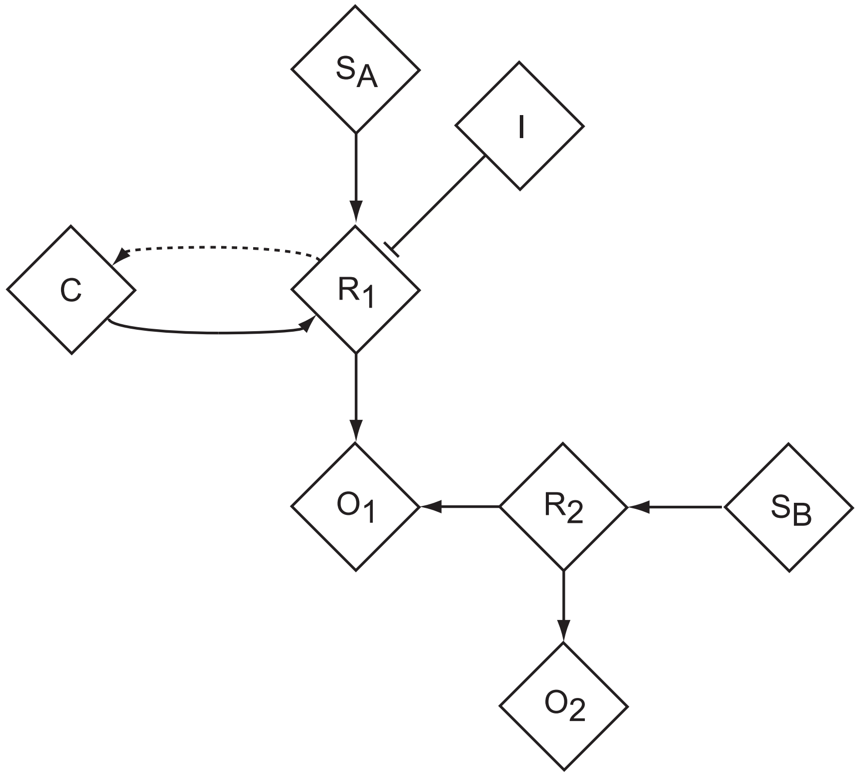

There is a strongly connected component (SCC) in this network consisting of $C \rightarrow R_1 \rightarrow C$, representing the catalytic action of C. The presence of the logic underlying the SCC can lead to erroneous predictions, and the system of logical statements must be modified appropriately. In this case, we can either delete the relationship $C \rightarrow R_1$, or $R_1 \rightarrow C$.

We must retain $C \rightarrow R_1$, as our formulation requires the presence of the catalyst in order for the signalling event to take place. The relationship $R_1 \rightarrow C$ (the dashed edge) represents redundant information — R_1 cannot be an active event unless C is already active — and is safe to remove. Not all curation issues are as clear as this example, and care must be taken when modifying the logical statements so as not to introduce funtion-altering modifications to the network.

The resulting system of production statements is:

$$\neg S_A \vee \neg C \vee I \rightarrow \neg R_1 \tag{16}$$

$$S_B \rightarrow R_2 \tag{17}$$

$$R_1 \vee R_2 \rightarrow O_1 \tag{18}$$

$$R_2 \rightarrow O_2 \tag{19}$$

1.7 Step 6: Conversion to Integer Constraints

Finally, the OR-form production rules are converted for solution in an integer program, again as per Haus (2009). For a system of production rules S of the form $L \rightarrow R$ where $L = \{x_{l_1}, x_{l_2}, \ldots x_{l_m}\}$ and $R = \{x_{r_1}, x_{r_2}, \ldots x_{r_n}\}$:

$$\sum_{0<i\leq m} x_{l_i} - x_{r_k} \geq 0 \text{ for all } S_i \in S, 0 < k \leq n$$

$$-x_{l_i} + \sum_{0<k\leq n} x_{r_k} \geq 0 \text{ for all } S_i \in S, 0 < i \leq m$$

$$x_l + \bar{x}_l = 1 \; l \in L$$

$$x_p = 1, x_q = 0 \; p \in L_1, q \in L_0$$

For this example, the resulting constraints are:

From statement 16:

$$(1 - x_{S_A}) + (1 - x_C) + x_I - (1 - x_{R_1}) \geq 0 \tag{20}$$

$$-(1 - x_{S_A}) + (1 - x_{R_1}) \geq 0 \tag{21}$$

$$-(1 - x_C) + (1 - x_{R_1}) \geq 0 \tag{22}$$

$$-x_I + (1 - x_{R_1}) \geq 0 \tag{23}$$

From statement 17:

$$x_{S_B} - x_{R_2} = 0 \tag{24}$$

From statement 18:

$$x_{R_1} + x_{R_2} - x_{O_1} \geq 0 \tag{25}$$

$$-x_{R_1} + x_{O_1} \geq 0 \tag{26}$$

$$-x_{R_2} + x_{O_1} \geq 0 \tag{27}$$

From statement 19:

$$x_{R_2} - x_{O_2} = 0 \tag{28}$$

System inputs for this network are S_A, C, I, and S_B. System outputs are O_1 and O_2.

2 Minimum Input

The goal of the minimum input problem is to find an assignment of variables to a set of system inputs ($ACTIVE$) that give rise to a user-specified model state $STATES$ so that the size of $ACTIVE$ is minimized. In the event that the user specifies a $STATES$ assignment that is not biologically feasible in the context of the signalling model M, then an error condition should be indicated.

Algorithm 1 Compute the set of inputs with minimal cardinality that results in a given network state.

Input: A tuple (O, B, V) describing the objective function O, set of bounds $B = \{b_1, b_2, \ldots b_n\}$, and set of variable states $V = \{v_1, v_2, \ldots, v_n\}$ of an integer programming problem LP. We assume that the constraints of LP have been set. Additionally, we require user-defined sets of variables identifying system input(s) ($INPUTS$), and variable assignments ($STATES = \{s_1, s_2 \ldots s_n\}$).

Output: A set of variable assignments to members of $INPUTS$, $ACTIVE$, such that the size of $ACTIVE$ is minimal and instantiating LP with $ACTIVE$ as the set of defined variables produces a set of variables $V = STATES$ after the model is solved.

$O \leftarrow \sum INPUTS$
for $i = 0$ to n **do**
  if $s_i =$ TRUE **then**
    $b_i \leftarrow (v_i = 1)$
  else if $s_i =$ FALSE **then**
    $b_i \leftarrow (v_i = 0)$
  else
    $b_i \leftarrow (0 \leq s_i \leq 1)$
  end if
end for
$LP \leftarrow (O, B, V)$
Solve LP for minimization of O
if LP was infeasible **then**
  return -1
else
  for variables v_i identified in $INPUTS$ **do**
    if $v_i = 1$ **then**
      Add active v_i to $ACTIVE$
    end if
  end for
  return $ACTIVE$
end if

---

3 Minimum Input Set

The minimum input sets problem is that of finding the set of distinct minimum inputs. Minimum inputs A and B are distinct if $A \backslash B \neq \varnothing$ and $B \backslash A \neq \varnothing$. The general approach to enumerating members of the minimum input set is outlined below:

Algorithm 2 Compute the minimum input set resulting in a given network state.

Input: LP, an integer programming solver with constraints, objective and bounds instantiated. $CUTS$, a set of variables in LP representing a set of integer cuts. $INPUTS$, a set of variables describing system inputs.

Output: A minimum input set containing all distinct minimum inputs.

```
for each variable v_i contained in CUTS do
  b_i ← (v_i = 0)
end for
Solve LP, minimizing the objective function
if LP is infeasible then
  Terminate this branch
else
  Store the minimum input generated by LP
  for each variable v_i contained in INPUTS do
    if v_i is active in the solution for LP then
      CUTS' ← a copy of CUTS
      Add v_i to CUTS'
      if CUTS' hasn't been processed before then
        Recursively compute the minimum input set using CUTS'
      end if
    end if
  end for
end if
return the stored minimum input set
```

Initially, this algorithm is invoked with LP established as in the minimum input problem and $CUTS = \varnothing$, to compute the minimum input giving rise to a user-specified model state.

Successive integer cuts are then applied in order to enumerate the set of all distinct minimum inputs. If a solution to LP has n inputs active, the number of possible immediate descendents is $2^n - 1$ (ie, cuts based on the powerset of active inputs, excluding the empty set). However, infeasibility due to cutting some variable v_i from the solution means that any problem where $v_i \in CUTS$ must also be infeasible, thus allowing us to create n descendents instead of $2^n - 1$ without loss of generality or exclusion of valid solutions.

4 Description of Data

4.1 Reactome

The Reactome data used in this study is dated 22nd September 2011 and comprises data for Homo sapiens in BioPAX Level 3 format sourced from Reactome.

Table 1: Pathways Removed from Reactome Data

Metabolism of amino acids and derivatives
Metabolism of carbohydrates
Metabolism of lipids and lipoproteins
Metabolism of nucleotides
Metabolism of porphyrins
Pyruvate metabolism and Citric Acid (TCA) cycle
Respiratory electron transport, ATP synthesis by chemiosmotic coupling, and heat production by uncoupling proteins
ABC-family proteins mediated transport
SLC-mediated transmembrane transport
HIV Infection

Table 2: Molecules Removed from Reactome Data

NAD cytosol	adenosine cytosol
proton cytosol	UBIQ_HUMAN nucleoplasm
NADH cytosol	diphosphate nucleoplasm
water extracellular region	GMP Golgi lumen
guanosine, deoxyguanosine cytosol	Adenosine 5'-triphosphate extracellular region
Phosphate cytosol	CO2 endoplasmic reticulum lumen
Mg++ cytosol	Cl- extracellular region
diphosphate cytosol	Cl- cytosol
Adenosine 5'-triphosphate cytosol	UDP endoplasmic reticulum lumen
ADP cytosol	Zn2+ extracellular region
Oxygen nucleoplasm	Ammonia cytosol
CO2 nucleoplasm	UDP cytosol
Guanosine 5'-triphosphate cytosol	NH4+ cytosol
Guanosine 5'-diphosphate cytosol	S-adenosylmethionine nucleoplasm
H2O cytosol	S-adenosylhomocysteine nucleoplasm
CoA-SH cytosol	Guanosine 5'-triphosphate plasma membrane
Acetyl coenzyme A cytosol	Guanosine 5'-diphosphate plasma membrane

NADPH cytosol	NADPH extracellular region
NADP+ cytosol	NADP+ extracellular region
H+ extracellular region	(d)NDP cytosol
Oxygen mitochondrial intermembrane space	(d)NTP cytosol
CO2 mitochondrial intermembrane space	K+ extracellular region
Adenosine 5'-triphosphate nucleoplasm	K+ cytosol
ADP nucleoplasm	Guanosine 5'-triphosphate nucleoplasm
Sodium extracellular region	GDP nucleoplasm
Sodium cytosol	hydrogencarbonate cytosol
S-adenosylmethionine cytosol	Acetyl coenzyme A nucleoplasm
S-adenosylhomocysteine cytosol	NADP+ peroxisomal matrix
NH4+ mitochondrial matrix	CO2 peroxisomal matrix
Hydrogencarbonate mitochondrial matrix	NADPH peroxisomal matrix
Adenosine 5'-triphosphate mitochondrial matrix	(d)GDP cytosol
Phosphate mitochondrial matrix	(d)GMP cytosol
ADP mitochondrial matrix	hydrogencarbonate extracellular region
NADPH mitochondrial matrix	NADPH nuclear envelope
Oxygen mitochondrial matrix	H+ nuclear envelope
H+ mitochondrial matrix	NADP+ nuclear envelope
H2O mitochondrial matrix	ADP peroxisomal membrane
NADP+ mitochondrial matrix	Phosphate peroxisomal membrane
(d)CMP, UMP cytosol	Oxygen stored secretory granule
(d)CDP, UDP cytosol	H2O stored secretory granule
TTP nucleoplasm	H+ mitochondrial intermembrane space
H+ early endosome lumen	(d)ADP, (d)CDP cytosol
(d)NDP mitochondrial matrix	Guanosine 5'-diphosphate mitochondrial matrix
Oxygen endoplasmic reticulum lumen	Guanosine 5'-triphosphate mitochondrial matrix
H+ endoplasmic reticulum lumen	CoA-SH endoplasmic reticulum lumen
NADPH endoplasmic reticulum lumen	Zn2+ cytosol
NADP+ endoplasmic reticulum lumen	inorganic phosphate extracellular region
H2O endoplasmic reticulum lumen	ADP, GDP, CDP, UDP cytosol
Calcium cytosol	H+ synaptic vesicle
Phosphate nucleoplasm	Cl- endoplasmic reticulum lumen
Oxygen cytosol	FAD mitochondrial inner membrane
CoA-SH peroxisomal matrix	FADH2 mitochondrial inner membrane
Acetyl coenzyme A peroxisomal matrix	Calcium plasma membrane
GMP cytosol	CMP, UMP cytosol
dNTP nucleoplasm	adenosine 5'-monophosphate peroxisomal matrix
Mg++ extracellular region	Calcium endoplasmic reticulum lumen
H2O nucleoplasm	NH3 nucleoplasm
Coenzyme A mitochondrial matrix	UDP nucleoplasm
NAD mitochondrial matrix	Phosphate endoplasmic reticulum lumen

CO2 mitochondrial matrix	(d)GMP, (d)IMP cytosol
NADH mitochondrial matrix	(d)CMP, TMP, (d)UMP cytosol
CO2 cytosol	Guanosine 5'-triphosphate endosome
ubiquitin cytosol	Guanosine 5'-diphosphate endosome
Acetyl coenzyme A Golgi lumen	H+ lysosomal lumen
NAD peroxisomal matrix	GMP, IMP cytosol
NADH peroxisomal matrix	ADP, GDP, CDP, UDP nucleoplasm
H+ peroxisomal matrix	dNTP cytosol
diphosphate mitochondrial matrix	Adenosine extracellular region
ADP extracellular region	Adenosine 5'-triphosphate endosome
GMP nucleoplasm	ADP endosome
Acetyl coenzyme A mitochondrial matrix	H+ late endosome lumen
(d)ADP cytosol	(d)AMP, (d)GMP, (d)IMP cytosol
(d)AMP cytosol	(d)A, (d)G, (d)I cytosol
Formic acid cytosol	UDP extracellular region
FAD mitochondrial matrix	Mg++ plasma membrane
FADH2 mitochondrial matrix	(d)CMP cytosol
Calcium extracellular region	Zn2+ endoplasmic reticulum lumen
H2O peroxisomal matrix	Zn2+ endosome lumen
Oxygen peroxisomal matrix	H2O mitochondrial intermembrane space
NH4+ peroxisomal matrix	Calcium Golgi lumen
H+ endosome lumen	CMP, TMP. UMP extracellular region
NH4+ extracellular region	H+ Golgi lumen
NTP nucleoplasm	Sodium Golgi lumen

4.2 Panther Pathways - Apoptosis and T-Cell Activation

Data from Panther Pathways for Apoptosis and T-Cell Activation used in this study is dated 9th November 2011. No molecules were removed from the Apoptosis data set. A table of molecules removed from the T-Cell Activation data follows:

Table 3: Molecules Removed from Panther Pathways T-Cell Activation Data

ADP
ADP Nucleus
ATP
ATP Nucleus
GDP
GTP
